# Mouse Mammary Tumor Virus Molecular Biology and Oncogenesis

**DOI:** 10.3390/v2092000

**Published:** 2010-09-23

**Authors:** Susan R. Ross

**Affiliations:** Department of Microbiology and Abramson Cancer Center, University of Pennsylvania, 421 Curie Boulevard, Philadelphia, PA 19104, USA; E-Mail: rosss@mail.med.upenn.edu; Tel.: +1-215-898-9764; Fax: +215-573-2028

**Keywords:** milk-borne virus, superantigen, intrinsic immunity, CIS, breast cancer

## Abstract

Mouse mammary tumor virus (MMTV), which was discovered as a milk-transmitted, infectious cancer-inducing agent in the 1930s, has been used since that time as an animal model for the study of human breast cancer. Like other complex retroviruses, MMTV encodes a number of accessory proteins that both facilitate infection and affect host immune response. *In vivo*, the virus predominantly infects lymphocytes and mammary epithelial cells. High level infection of mammary epithelial cells ensures efficient passage of virus to the next generation. It also results in mammary tumor induction, since the MMTV provirus integrates into the mammary epithelial cell genome during viral replication and activates cellular oncogene expression. Thus, mammary tumor induction is a by-product of the infection cycle. A number of important oncogenes have been discovered by carrying out MMTV integration site analysis, some of which may play a role in human breast cancer.

## 1. Introduction

Non-acute transforming retroviruses, which cause dysregulated expression of cellular oncogenes upon integration of the provirus into the host genome, have long been used to study both tumor development and progression in animal models [1]. Indeed, many oncogenes were originally identified because of their association with tumors caused by this type of retrovirus. Although the majority of non-acute transforming retroviruses induce cancer in cells of hematopoietic origin, one exception is the murine betaretrovirus MMTV, which causes mammary epithelial cell tumors. MMTV was first characterized in the 1930s as a milk-transmitted agent associated with mammary tumors in mice [2] and thus has long been used as an *in vivo* model for the study of human breast cancer [3]. Here, I briefly review MMTV molecular biology, its *in vivo* infection pathway, and what has been learned about how this virus causes mammary tumors.

## 2. MMTV genome and proteins

MMTV was originally classified as a simple retrovirus. The genomes of simple retroviruses encode the virion capsid/nucleocapsid (Gag) proteins, the enzymes needed for genome replication (reverse transcriptase and integrase; Pol/In) and the envelope proteins (Env) that bind the cell surface molecule(s) used for virus entry [1]. In contrast, the genomes of complex retroviruses, such as lentiviruses and deltaviruses, encode non-structural proteins that facilitate virus replication or that counteract intrinsic, innate or adaptive immune responses during *in vivo* infection. Because the MMTV genome encodes at least three known accessory proteins that carry out these functions, it is now considered a complex retrovirus.

**Figure 1.**
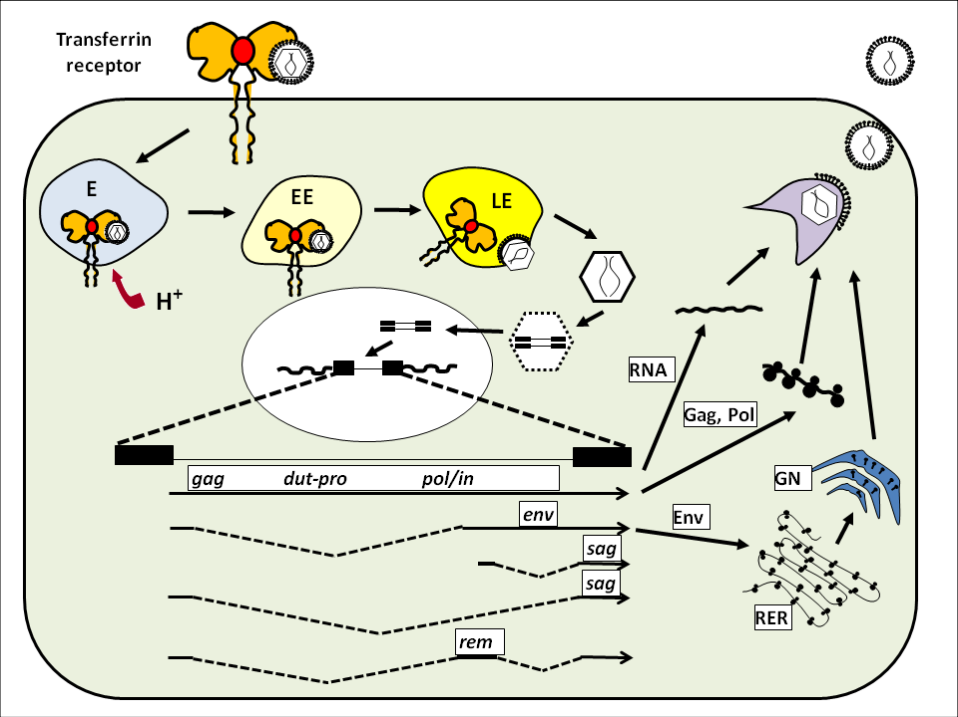
MMTV infection. MMTV binds to TfR1 on the cell surface and is internalized into a low pH compartment. After uncoating, the viral genome is reverse-transcribed, is transported to the nucleus, and the provirus integrates into the genome. At least five different RNAs are transcribed from the integrated provirus (see text). The Env membrane proteins are synthesis in the rough endoplasmic reticulum (RER) and traffic through the Golgi network (GN). Little is known about MMTV virion assembly, although it occurs in an intra-cellular compartment. Abbreviations: E, endosome; EE, early endosome; LE, late endosome.

The MMTV is 9 kb in length and like all retroviruses, is flanked by 5′ and 3′ long terminal repeats (LTRs), which in the case of MMTV are exceptionally long (approximately 1.3 kb). This is because the MMTV 3′ LTR encodes one of the viral accessory proteins, termed the superantigen (Sag) (Figure 1). In addition to the Sag coding region, there are a number of transcription factor binding sites in the LTR that determine tissue-specific, as well as glucocorticoid/progesterone-regulated virus expression [4–6]. Because they direct high level, hormone regulated expression in mammary epithelial cells, the MMTV LTRs have been extensively used to drive oncogene expression in transgenic mice as a means of creating mouse models of breast cancer [3]. Similarly, mammary gland-specific deletion of tumor suppressor genes using MMTV LTR-cre recombinase transgenic mice has resulted in additional breast cancer models [7].

MMTV also infects lymphocytes *in vivo* (see next section) and the MMTV LTRs contain regulatory elements that control expression in B and T cells [8,9]. Thus, MMTV-oncogene transgenic mice frequently develop lymphoid as well as mammary cell tumors (for example, see [10]). Although MMTV infects and is expressed in lymphocytes, the virus usually only causes mammary tumors. However, there are MMTV variants with deletions/insertions in the LTRs that are causally associated with T cell lymphomas; the sequence alterations in the LTR are believed to result in the generation of transcriptional enhancers that activate linked cellular oncogene expression in these cells [11,12].

At least five transcripts are generated from the MMTV genome. A full-length, unspliced RNA that initiates in the 5′ LTR and terminates in the 3′ LTR serves as the viral genome and is packaged into virions [1]. This transcript also functions as the mRNA for the Gag and Pol proteins, as well as a dUTPase (Dut) and other proteins of unknown function (Figure 1). The role of the dUTPase in MMTV infection is not known, but the Dut of the lentivirus equine infectious anemia virus (EIAV) is thought to facilitate replication of EIAV in non-dividing cells by maintaining adequate nucleotide pools [13].

The Env protein, which mediates retroviral entry into cells by binding specific cell surface molecules, is encoded by a singly spliced mRNA (Figure 1). As with other retroviruses, the MMTV Env consists of two chains, generated by cellular furin processing of a 73 kD polyprotein precursor to a 52 kD cell surface (SU) protein, which binds the entry receptor, and a 36 kD transmembrane (TM) domain that is responsible for membrane fusion. MMTV uses transferrin receptor 1 (TfR1) for entry [14]. TfR1, the major means by which cells take up iron [15], traffics to the acidic endosome upon ligand (iron-bound transferrin) binding. MMTV entry requires low pH (<5.2) and thus, most likely co-traffics with TfR1 to a late endosomal compartment after binding at the cell surface [14,16] (Figure 1). TfR1 is highly expressed on activated lymphocytes and dividing mammary epithelial cells *in vivo*, which probably limits MMTV infection to these cell types [17–19]. Additionally, as described above, the transcriptional regulatory elements in the MMTV LTR function predominantly in mammary epithelia and lymphoid cells, thus further restricting infection and spread to other tissues *in vivo*.

At least two additional accessory genes, Sag and Rem, are encoded by other alternatively spliced mRNAs (Figure 1). The Sag protein is translated from two different transcripts (Figure 1). The first transcript initiates in the 5′ LTR and uses the same splice donor as the *env* mRNA. The second transcript uses a promoter and splice donor in *env*. Both transcripts use a splice acceptor just upstream of the open reading frame in the 3′ LTR [20–22]. The regulator of export of MMTV (Rem) protein is encoded by a double spliced mRNA [23,24]. Like the human immunodeficiency virus (HIV) Rev protein, Rem is required for efficient transport of unspliced viral RNA from the nucleus through interaction with a Rem-responsive element present in the MMTV RNA [25,26].

As is the case for other betaretroviruses, MMTV virion assembly occurs in an intracellular compartment and virions are then exported to the plasma membrane for egress from the cell [27]. However, little is known about either the viral or cellular requirements for MMTV assembly. *In vivo*, virus is shed from the apical surface of polarized mammary epithelial cells into the lumen of the end buds, along with the milk proteins and thus is transmitted to offspring [28].

## 3. MMTV *in vivo* infection

MMTV is transmitted from infected mothers to pups through nursing, most likely as cell-free virus which is present at very high levels in milk [29]. Susceptible strains that acquire exogenous MMTV through milk can be freed of the virus by foster-nursing on uninfected mothers. Viruses isolated from mice that shed milk-borne MMTV infect and cause tumors in different strains of mice to varying degrees [30]. This differential susceptibility to MMTV infection is due in large part to intrinsic, innate and adaptive immune responses to the virus that vary between mouse strains (see below). In addition to the exogenous form of MMTV, all commonly used inbred mice as well as many wild mice inherit endogenous copies of MMTV which are thought to have entered the mouse genome between 10–20 million years ago [31]. The vast majority of endogenous MMTVs do not produce infectious virus due to deletions or mutations in the proviral genome. At least 10 different exogenous and greater than 30 endogenous MMTVs have been identified (for example, see [32–35]).

With the exception of the virions found in milk, exogenous MMTV in infected mice is cell-associated [36] and infection of lymphocytes is critical for virus spread from the gut to the mammary tissue (reviewed in [37]). In brief, MMTV acquired through milk first infects dendritic cells (DCs) then spreads to T and B cells in the Peyer’s patches of the gut and ultimately to other lymph nodes and lymphoid organs. This spread is largely due to the viral Sag protein, which is presented to the T cell (TCR) on CD4+ T cells by major histocompatibility (MHC) class II proteins expressed on the surface of infected antigen presenting cells, such as DCs and B cells. The Sag-activated T cells proliferate, provide B cell help and produce cytokines that stimulate and recruit additional DCs, B and T cells, resulting in the establishment of a reservoir of infection-competent and infected lymphocytes. The mammary gland is considered mucosal-associated lymphoid tissue (MALT) and represents a site of normal lymphocyte trafficking. MMTV-infected lymphocytes are therefore not only an important reservoir for amplification of the virus *in vivo*, but also carry virus to the mammary gland [38,39].

MMTV infects mammary epithelial cells at a time when they are driven to divide, that is, during the cell division that accompanies both puberty and pregnancy. Mice that go through multiple rounds of pregnancy have much higher levels of MMTV infection in mammary tissue [36]. It is believed this is due to the increased susceptibility of dividing mammary epithelial cells to infection by lymphocyte-produced virus and not to horizontal spread of the virus between mammary cells [38]. Transcription of viral RNA and production of virus is highest during late lactation and pregnancy, thereby maximizing virion production in milk.

Different exogenous and endogenous MMTVs encode Sag proteins with different TCR specificity. *Sag*s encoded by endogenous MMTVs cause the deletion of cognate T cells during shaping of the immune repertoire, while those encoded by exogenous virus produce a slower but none-the-less almost complete deletion of such lymphocytes [40]. This results in mice that are immune to infection by exogenous MMTVs with the same Sags because they lack responding T cells [41]. MMTV Sag-induced T cell deletion may also make some mouse strains more resistant to the pathogenic effects of Leishmania major because of loss of T cell responses [42]. Interestingly, while most of the endogenous MMTVs sustain mutations in the coding regions for the virion proteins, almost all retain intact Sag coding regions, suggesting that the anti-viral and -parasite protection might confer a selective advantage to mice that retain endogenous MMTVs.

There are other host genes that confer resistance or susceptibility to MMTV. These include MHC Class II genes required for efficient Sag presentation, the innate immune pathogen sensor toll-like receptor 4 (*Tlr*4), and several as-of-yet unidentified genes that affect the production of anti-MMTV antibodies or T cell responses to MMTV (reviewed in [43]). MMTV infection is also restricted by the intrinsic immune factor, apolipoprotein B editing complex 3 (APOBEC3) [44]. Different inbred mouse strains have polymorphisms in *Apobec*3 [45], which affect the ability of this factor to inhibit MMTV infection [46]. All of the host genes that affect MMTV lymphocyte infection also alter the incidence and kinetics of mammary tumor induction. Interestingly, host genes that affect mammary tumorigenesis do so by limiting virus infection; none have been shown to have a direct effect on the ability of the virus to transform mammary epithelial cells.

## 4. MMTV and Mammary Tumorigenesis

MMTV-induced mammary tumorigenesis is mediated by proviral integration, usually by enhancer -mediated activation of nearby cellular oncogenes, although a few examples of coding region insertions that alter the gene product have also been described [47]. Additionally, the MMTV Env protein has been implicated in mammary tumorigenesis. The MMTV Env has an immuno-tyrosine based activation motif (ITAM) commonly found in receptors expressed in hematopoietic cells; expression of the Env protein in normal mouse or human mammary epithelial cells induces morphological transformation in culture [48]. Moreover, when the ITAM motif was mutated in the virus, mammary tumor-induction was attenuated in infected mice [49].

*In vivo* infection levels usually affect mammary tumor incidence and kinetics because of an increased number of proviral integrations. A number of different common integration sites (CIS) have been implicated in MMTV-induced mammary tumors (Table 1), particularly the *Wnt* and *Fgf* genes [47]. MMTV-induced tumors appear to develop from infection of a single stem cell [50]. Mammary stem cells have the potential to form two epithelial lineages in the mammary gland, myoepithelial cells found on the outside of ducts, and ductal and alveolar luminal cells. MMTV-induced tumors proceed through at least two stages, the pregnancy-dependent hyperplastic alveolar nodule (HAN), followed by the hormone-independent mammary tumor [51]. Although the histopathological features of MMTV-induced tumors do not usually resemble the most frequent forms of human breast tumors such as invasive ductal carcinomas, many, if not all, of the human mammary lesions are thought to originate in the terminal ductal lobular unit and atypical lobular type A lesions (ALA) are morphologically similar to the mouse mammary HAN lesions [51].

Most MMTV-induced mammary tumors contain 10 or more proviral integrations [51]. Whether MMTV-induced mammary tumors are the result of a single initiating integration or are a composite of multiple integrations is currently unclear, although it appears that progression of tumors from a pregnancy-dependent to a pregnancy-independent state depends on additional integration events and that the former frequently arise as polyclonal populations while the latter are generally monoclonal [34,52]. That mammary tumorigenesis requires the activation of multiple oncogenes is supported by several observations. First, a large percentage of mammary tumors derived from MMTV-infected wild type mice have “hits” at both *Wnt*1 and *Fgf*3; it is likely that additional genes are activated in these tumors. Second, the creation of double transgenic mice, such as MMTV-Wnt1/MMTV-Fgf3 accelerates the induction of mammary tumors [53–55]. Similarly, tumorigenesis is accelerated by MMTV infection of transgenic mice with genetic predisposition to mammary tumorigenesis, such as those that express the Wnt1, c-neu or TGFβ transgenes in mammary tissue [54,56].

The oncogenes listed in Table 1 were identified by infection of both wild type and transgenic mice expressing oncogenes in mammary tissue. Although most of the CIS listed in Table I were identified through low throughput analyses, more recently, high throughput analysis of MMTV insertion sites has been performed and uncovered gene families not previously associated with cancer [66]. MMTV target site analysis has great potential for uncovering pathways relevant to the human disease, particularly in transgenic strains with tumor morphology similar to that seen in humans. Even though MMTV-induced mammary tumors do not morphologically resemble most human breast cancers, there are molecular similarities between the mouse and human disease. For example, although *Wnt*1 itself has not been implicated in human breast cancer, other members of this family as well as targets of the Wnt signaling pathway, such as β-catenin, E-cadherin, cyclin D1, *etc.,* are known to be mutated or deregulated in a number of human cancers [67,68] and several *Fgf* family members have been implicated in human breast cancer [59,69]. Moreover, a number of the novel CIS uncovered in the high throughput screens were found to be dysregulated in human breast cancer and correlated with one or more clinical parameters in the human tumors, such as angioinvasion, tumor grade or lymphatic infiltration, as well as metastasis [66].

Since the discovery of MMTV, numerous investigators have searched for a similar virus in human breast cancers (see [70] and [37] for a more extensive review). Early studies using immunological assays detected antibodies against MMTV proteins in sera from human breast cancer patients, but were later shown to be most likely due to non-specific cross-reactive proteins [71]. Molecular analyses for DNA sequences related to MMTV in human DNA resulted in the discovery of the first human endogenous retroviruses (HERVs) because of their homology to MMTV, particularly in the *pol* gene, but no MMTV-like sequences were identified [72]. More recently, several groups have used polymerase chain reaction (PCR)-based techniques to amplify MMTV-like sequences from human breast cancer biopsies [73,74]; however, others have been unable to replicate these studies [75–78]. Moreover, it has been reported that MMTV can infect cultured human mammary epithelial cells, but MMTV cannot use human transferrin receptor 1 as an entry receptor and thus would have to infect cells via a different entry mechanism [14,16,79]. Thus, the existence of a human MTV remains to be confirmed.

## 5. Conclusions and perspectives

Much has been learned about *in vivo* virus infection using MMTV and mice. This is in a large part due to our ability to study the natural route of milk-borne transmission and to the use of both inbred and genetically altered transgenic and knockout mice in the analysis of infection and oncogenesis. Because MMTV has existed as an infectious virus in mice for millions of years, it has evolved to take advantage of its host’s biology, using host genes from transcription factors to immune regulatory molecules, to establish infection. Although it causes mammary tumors, this does not occur until relatively late in life and thus the virus has persisted, since infected mothers are able to transmit virus to offspring. The lack of acute MMTV-induced pathogenesis is most likely due to different host means of limiting virus infection, including factors that operate at the cellular level like intrinsic restriction factors and immune response genes. As additional host-anti-viral genes are discovered, MMTV will continue to serve as an important model for testing the ability of these factors to function *in vivo*.

In addition to serving as an important means for studying virus infection, MMTV has provided a number of critical models for understanding human breast cancer. First, the analysis of MMTV CIS has led to the identification of oncogenes/pathways such as members of the *Wnt* family that are known to play roles in different human cancers. Second, ITAM-mediated signaling in mammary epithelial cells may represent a novel mechanism of transformation; indeed, a novel oncogene associated with invasive breast cancer in humans has been recently shown to transform cells through ITAM-mediated signaling [80]. Finally, the use of the MMTV LTR to direct oncogene expression to murine epithelial cells has resulted in the creation of numerous transgenic mouse strains that serve as critical models for understanding human breast cancer. It is likely that such transgenic mice will continue to be a critical tool as additional human breast cancer genes are identified through large-scale human genetic studies.

## Figures and Tables

**Table 1.** MMTV common integration sites.

**Mouse**	**Oncogene**	**Reference**
Wild type	*Wnt*1/*Wnt*10b	[57]
Wild type	*Fgf*3	[58]
Wild type	*Fgf*10	[59]
MMTV-*Wnt*1	*Fgf*8	[55]
Wild type	*Notch*4	[60]
Wild type	int-5/aromatase	[61]
MMTV-neu	*Notch*1	[56]
WAP-TGFβ	*Wnt*1/*Wnt*3	[62]
Wild type	eIF3e-p48	[63]
Wild type	*Rspo*2	[64]
Wild type	*Rspo*3	[65,66]
